# Working among the rural communities in Ghana - why doctors choose to engage in rural practice

**DOI:** 10.1186/s12909-018-1234-y

**Published:** 2018-06-08

**Authors:** Anthony Amalba, Francis A. Abantanga, Albert J. J. A. Scherpbier, W. N. K. A. van Mook

**Affiliations:** 1 Department of Health Professions Education and Innovative Learning, School of Medicine and Health Sciences (SMHS), University for Development Studies (UDS), P. O. Box, 1883 Tamale, Ghana; 2 Faculty of Health, Medicine and Life Sciences, School of Health Professions Education, Maastricht University, Maastricht, the Netherlands

**Keywords:** Community-based education, Medical doctors, Rural community, Rural practice

## Abstract

**Background:**

An unequal distribution of health personnel, leading to unfavourable differences in health status between urban and rural populations, is a serious cause for concern globally. Part of the solution to this problem lies in attracting medical doctors to rural, remote communities, which presents a real challenge. The present study therefore explored the factors that influence medical doctors’ decision to practise in rural Ghana.

**Methods:**

We conducted a cross-sectional descriptive study based on questionnaires. Participants were doctors working in health facilities in the districts and rural areas of the Northern Region, Ghana. The qualitative data analysis consisted of an iterative process of open, axial and selective coding.

**Results:**

We administered the questionnaires to 40 doctors, 27 of whom completed and returned the form, signalling a response rate of 67.5%. The majority of the doctors were male (88.9%) and had been trained at the University for Development Studies, School of Medicine and Health Sciences (UDS-SMHS) (63%). Although they had chosen to work in the remote areas, they identified a number of factors that could prevent future doctors from accepting rural postings, such as: a lack of social amenities, financial and material resources; limited career progression opportunities; and too little emphasis on rural practice in medical school curricula. Moreover, respondents flagged specific stakeholders who, in their opinion, had a major role to play in the attraction of doctors and in convincing them to work in remote areas.

**Conclusions:**

The medical doctors we surveyed had gravitated to the rural areas themselves for the opportunity to acquire clinical skills and gain experience and professional independence. Nevertheless, they felt that in order to attract such cadre of health professionals to rural areas and retain them there, specific challenges needed addressing. For instance, they called for an enforceable, national policy on rural postings, demanding strong political commitment and leadership. Another recommendation flowing from the study findings is to extend the introduction of Community-Based Education and Service (COBES) or similar curriculum components to other medical schools in order to prepare students for rural practice, increasing the likelihood of them accepting rural postings.

**Electronic supplementary material:**

The online version of this article (10.1186/s12909-018-1234-y) contains supplementary material, which is available to authorized users.

## Background

The unequal global distribution of health personnel is a serious problem confronting the health sector. This disparity of health personnel between the rural and urban populations can contribute to differences in health outcomes [1]. People living in rural communities are not well educated, poorer and have the worst access to healthcare, compared to those in urban areas [2]. In fact, in proposing his inverse care law, Hart already pointed out long before that those who have the greatest needs regarding healthcare normally have the worst access to healthcare services [3]. The absence of better living conditions or social amenities such as good schools with qualified teachers for children, good accessible roads and transportation system, electricity and potable water have been quoted internationally [4, 5] as barriers to the retention of health workers, notably doctors, in remote areas.

Some middle- and low-income countries have implemented several strategies to motivate health workers to accept rural postings. Thailand and Sierra Leone, for instance, have adopted coercion and financial measures to make doctors accept postings in rural areas. Likewise, the Ghanaian government has resorted to a 20–30% salary top-up and a staff vehicle hire purchase scheme for health staff in an effort to attract and retain health workers in rural areas. However, neither of these has yielded the desired results in addressing the lack of health professionals in rural areas. A review of the literature on the attraction of health staff to rural areas in middle- and low-income countries and their retention clearly points to poor working conditions such as a lack of potable water, poor sanitation, limited career progression prospects, a lack of management and community support and the absence of proper equipment and infrastructure at the health facility level [1, 6–9] as reasons deterring doctors from gravitating to rural areas. Other factors potentially influencing health workers’ willingness to practise in remote communities, as studies in the field of primary care have revealed, are socioeconomic status, rural background, gender, culture, and individual and curriculum characteristics, although some variations may occur across settings [8, 10–12].

Recognising that the shortages and maldistribution of the health workforce pose a significant threat to the health system in Ghana [13], the Ghanaian Ministry of Health has identified key areas that require attention in order for health workers to gravitate to the country’s rural areas and remain there, specifically: income disparity, lack of career progression opportunities, accommodation and workload [14, 15]. While previous research has addressed incentives for medical students to take up rural practice later in life, few studies have hitherto explored the factors that induce medical doctors in particular to gravitate (or not) to the rural areas of Ghana and remain there [14]. This is precisely what the present study seeks to accomplish.

## Methods

### Setting

As one of the developing countries in sub-Saharan Africa, Ghana is located on the West African coast and has a population of approximately 28 million people. We conducted this study in one of the country’s ten administrative regions, specifically the Northern Region, which has Tamale as its capital city and a population of over 2 million inhabitants. Most of the communities in the region are deprived. The region counts 345 health facilities and is divided into 26 districts, each having at least one district hospital, a number of health centres, Community-based Health Planning and Services (CHPS) compounds and maternity homes. The Ministry of Health has assigned specific levels of care to these facilities, depending on the category of health staff managing the facility: Level M refers to maternity homes (managed by midwives); Level A to CHPS compounds (managed by a nurse with public health background); Level B1 to health centres without a doctor; Level B2 to health centres with a doctor; Level C to District hospitals; and Level D to regional and tertiary hospitals (MOH, 2010).

The School of Medicine and Health Sciences of the University for Development Studies (UDS-SMHS) is the only medical school in the region that offers community training as an integral part of the curriculum, known as Community-Based Education and Service (COBES). Through COBES, students spend 4 weeks in the same community in each of the first 3 years of the programme. In the first year, they learn to identify and explain factors -demographic, social, environmental and economic- affecting the community’s health. In the second year, they conduct a study into the health needs resulting in a community health diagnosis, whereas in the third year they undertake to resolve those needs by identifying resources available in the community. The aim of this curriculum element is to teach students to provide service to the people in the community. In later years of their medical training, students are sent to the district hospitals for a community rotation of 6 to 8 weeks.

### Questionnaire and participants

We conducted a cross-sectional descriptive study among doctors working in health facilities in the Northern Region between September 2016 and March 2017. To collect data, we used a 23-item questionnaire consisting of both open- and closed-ended questions about participants’ demographic characteristics, medical school, motivation, challenges, perceptions and preferences related to work in a rural area (See Additional file 1). The questionnaire was developed by the first author, construct-validated by the second, third and fourth authors who reformulated some questions for clarity, and then pretested on three medical doctors in the West hospital, Tamale, who made minor changes. Participants were purposefully and conveniently selected among physicians in the district hospitals and level B2 health centres. Two research assistants visited all the district hospitals and level B2 health centres and distributed the questionnaires to all 40 doctors on the spot on the day of the visit. They explained the purpose of the study to participants and assured them of confidentiality of their responses. In follow-up visits, they collected the completed questionnaires. Participation was voluntary and all participants who agreed to participate in the study gave written informed consent. The Tamale Teaching Hospital Ethics Review Committee (TTHERC ID No. TTHERC/17/11/16/03) granted ethical approval for all data collection procedures and processes of obtaining informed consent. The Regional Director of Health Service, Northern Region, granted permission for the use of the district hospitals and health centres as study sites.

### Data analysis

We performed a qualitative content analysis of the responses to the open-ended questions using Atlas TI, version 6.0.15 GmbH-Berlin, and applying the principles of primary, secondary and tertiary coding [16]. AA and WvM read the responses independently, identifying common themes, trends and shared opinions among participants using content analysis. The identified themes were independently coded, enabling us to compare doctors’ responses. The independent codes generated by AA and WvM were cross-checked by the second and third author (FAA and AS). In the process, researchers discussed all discrepancies until they reached consensus.

## Results

In the following, we will first present the quantitative results, followed by the qualitative results pertaining to the open-ended questions. Illustrative quotes are cited where necessary.

### Quantitative results

Of the 40 doctors we invited, 27 completed the questionnaire, signalling a response rate of 67.5%. As shown in Table 1, the majority of the doctors working in the district and rural areas of northern Ghana were male (88.9%) and had been trained at the UDS-SMHS (63%).

**Table 1:** Participants’ demographic characteristics, including the no. of years spent in rural practice and the type of medical education received

	Number	Percent	
Gender			
Male	23	88.9	
Female	4	11.1	
Age (in years)^a^			
25–29	7	25.9	
30–34	9	33.3	
35–39	4	14.8	
40–44	3	11.1	
45–49	0	0	
50–54	2	7.4	
University attended			
UGSMD	2	7.4	
KNUST-SMS	1	3.7	
UDS-SMHS	17	63	
Others			
Eastern Europe	5	18.5	
Turkey	2	7.4	
Experience in rural practice (in years)			
0–11 months	9	33.3	
1–5 years	11	40.7	
Above 5 years	7	26	
Curriculum type^b^			
UGSMD			Traditional
KNUST-SMS			Traditional
UDS-SMHS			PBL/COBES
Eastern Europe			
Turkey			

^a^Two missing values

^b^UGSMD: University of Ghana School of Medicine and Dentistry; KNUST-SMS: Kwame Nkrumah University of Science and Technology-School of Medical Sciences; UDS-SMHS: University for Development Studies-School of Medicine and Health Sciences

As can be construed from Table 2 below, 74% of participants felt their respective medical schools had adequately prepared them for rural practice and that these schools were generally well-equipped to prepare medical students for this job.

**Table 2:** Participants’ perceptions of preparedness, awareness of the challenges involved and motivation to work in rural areas

Item	Yes		No	
	n	%	n	%
Medical school prepared^a^ me for rural practice	20	74.1	7	25.9
Medical school can prepare students for rural practice	20	74.1	7	25.9
Medical students are aware of the challenges				
related to living and working in rural areas^b^	20	76.9	6	23.1
Has your motivation to work in rural areas				
changed since you started working there?^b^	5	19.2	21	80.8

^a^Preparedness was measured by the number, if any, of community-based-education components in the curriculum

^b^One missing value

### Qualitative results

**Fig. 1:**
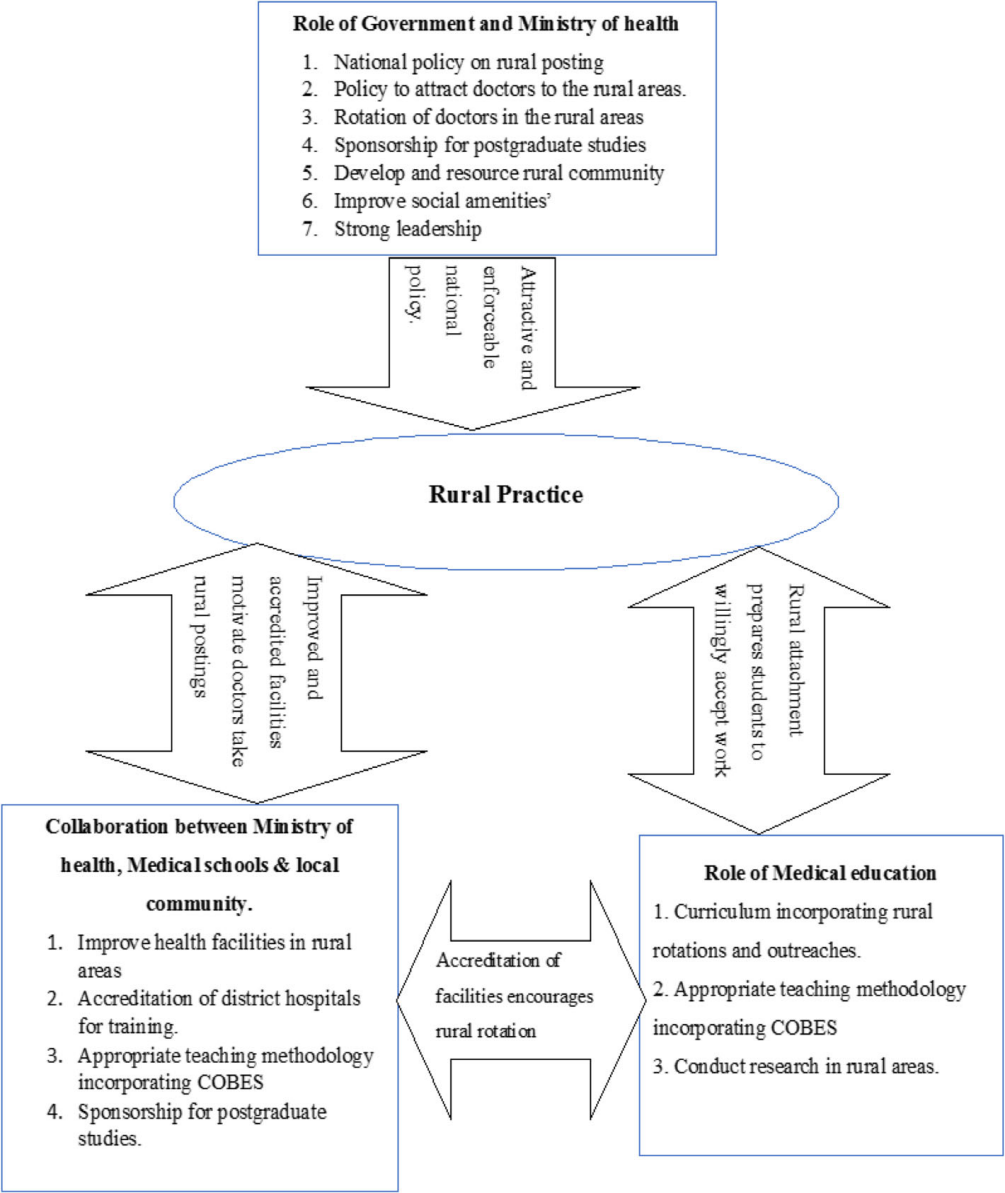
Strategies to encourage rural practice

In the following paragraphs, we will expand on each of the themes that emerged from the qualitative data analysis. For an overview of the main categories, subcategories and themes, please refer to Table 3. After that, we will highlight the key players who, in participants’ view, can make a major contribution to the attraction and retention of doctors in Northern Ghana’s rural areas (also see Fig. 1). In order to guarantee confidentiality and participants’ anonymity, we de-identified all quotes by assigning generic codes for both participants and institutions. While we numbered the health facilities (as in HF1 = Health Facility one), we also distinguished between male and female doctors (MD = male doctor; FD = female doctor). For example, a quote ending in HF2FD refers to a quote from a female doctor at health facility 2.

**Table 3:** Deterrents and motivators regarding practice in rural areas

Category	Sub categories	Themes
Motivators	Knowledge improvement	To acquire skills, knowledge and experience
		To become professionally independent
		Change of environment
	Altruistic considerations	Serving the needy is satisfying
		Awareness of inadequate human resources
Deterrents	Social challenges	Lack of social amenities
		Inadequate infrastructure of health facilities
	Financial challenges	Lack of financial and material resources
		Lack of tools and logistics
	Educational challenges	Little attention to rural practice in medical school curriculum
		Lack of skills training
		Teaching mostly theoretical
		Few career progression opportunities
		Lack of support from authority
		Lack of mentors
	Cultural challenges	Cultural barriers
		Family issues

### Motivators

#### Improvement of knowledge and skills

The vast majority of respondents were drawn to rural practice by the desire to acquire more clinical skills and experience. They anticipated that by working in rural practice they would gain more confidence and, consequently, they would become more professionally independent. In the words of one participant: *‘Having exposure to more clinical cases in the district and learning more surgical skills perceived to be available in the district’. HF1MD.*

#### Altruistic considerations

Most of the doctors have had earlier encounters with the health facilities in the rural areas during medical training, where they witnessed a heavy workload with a low doctor-patient ratio. Awareness of these deprivations, such as a lack of doctors, instilled in them the desire to give something back to the communities where they were trained. As a result, they decided to return to these communities upon completion of medical training. The following quote clearly illustrates this: *‘I visited the facility and saw the doctor-to-patient ratio was terrible with a variety of illnesses in the area. I realised the people needed help with more doctors hence I decided to stay’. HF10FD.*

### Deterrents

#### Social barriers

Although most of the doctors had gravitated to remote areas on their own free will, they did identify a number of barriers that could potentially deter future doctors from accepting rural postings (Table 3). Specific social barriers they mentioned were: a lack of good schools for their children, poor road networks and transportation, poor accommodation, poor Internet facilities and accessibility, and limited recreational activities. As one participant explained: ‘*Access to potable drinking water, electricity and other social amenities. Accommodation and other job opportunities are lacking in the rural area as compared to what pertains in the cities. Bad roads and poor transportation system’. HF14MD.*

#### Access to resources

A second factor that could keep doctors from settling and staying in rural areas was the undersupply of adequate tools, and financial and material resources in most of the facilities. Especially, the lack of basic work instruments and equipment frustrated most of the doctors who worked in the rural areas. Increasing the supply of such basic necessities could be a powerful strategy to attract doctors to the rural communities, as one of the participants suggested: ***‘****Facilities in rural areas should be equipped with basic logistics to make work easier’. HF13MD. And: ‘Lack of some basic and essential equipment in the district hospitals leading to otherwise unnecessary referrals’. HF6MD.*

#### Educational challenges

Some of the doctors pointed to the need for a greater emphasis on rural practice in medical training by offering rural rotations as an integral part of the curriculum. They experienced difficulties in finding a replacement when they wished to pursue their studies elsewhere in order to progress in their career. In their view, this was down to a lack of awareness among potential candidates of the benefits of rural practice. Consequently, doctors often felt neglected and abandoned by management or the authorities:

*‘Time should be devoted in the medical curriculum to create awareness to medical students as to the challenges involved in living and practising in rural areas and intensifying district rotations’. HF5MD.*

*‘It is difficult getting a doctor to take your place when the time is due for you to go for studies’, HF4FD.*

*‘Health workers in rural areas are always burdened and feel forgotten or neglected by the health authorities’. HF4MD.*

#### Family and cultural related factors

In a similar vein, cultural differences, language barriers, being unprepared for life in remote areas and being isolated from families could act as deterrents to practice in rural areas:

*‘Cultural practice, the challenges of rural lifestyle and the urge of living closer to my family make it difficult to accept to work in the rural area’. HF5FD.*

### Key players and their roles in addressing the undersupply of rural doctors

Participants flagged key players such as the Ghanaian Government, Ministries of Health and Education, medical schools and the community themselves, who had a major role to play in the attraction of medical officers to rural areas. Figure 1 provides an overview of these actors and their relevant roles. The next sections will elaborate on each of these actors and their roles which, for ease of understanding, have been translated into strategies to address the undersupply of doctors in rural and remote areas.

#### The government and ministries of health and education

##### Reinvigorate policies to attract doctors to rural areas

Participants argued that the power to attract more doctors to the rural areas was concentrated, in part, in the hands of the national authorities who could reinvigorate existing policies to attract and retain doctors in the rural areas in Ghana’.

*A good package and committed incentives such as rural service allowances which are at least 20% of their basic salary and reducing the duration for waiting to be promoted to the next rank to at least 3 years will help boost morale and encourage the doctors to want to serve in the rural areas. HF4MD.*

##### Sponsoring of postgraduate training in rural areas

Another suggestion made by participants was to offer sponsorships to medical graduates wishing to pursue their postgraduate training in rural areas: *‘Yes, the government can encourage doctors to serve rural Ghana by giving postgraduate sponsorships and other preferential treatment to doctors who work in rural Ghana’. HC3MD.*

##### Rotation of doctors in the rural areas

Some of the doctors suggested that a policy on compulsory rural rotation would help address the disparity of doctors between the rural and urban areas. They believed that such a strategy would afford young doctors the opportunity to learn to appreciate the rural lifestyle, potentially eliminating negative stereotypical views and inducing them to stay:

*If a policy can be put in place such that doctors are made to rotate through rural areas after house job for a fixed number of years, say 2 years, after which they can leave to any other facility of their choice if they wished, and be replaced by another medical officer. HF8FD.*

#### Medical schools

##### Integration of rural rotations/outreaches into the medical curriculum

Another group of actors perceived to wield the power to address the undersupply of rural doctors were medical schools. Participants felt strongly that immersion of students in rural areas should not only constitute an integral part of the curriculum but also, when present, be of significant duration. In their view, such incorporation of rural outreach/practice into the curricula would acquaint medical students with rural practice prior to graduation, thereby increasing the likelihood of them accepting rural postings. As the following participants pointed out: *‘There were no rural training courses in our curricula. Medical school should design or include as part of the training a rural outreach programme; this will help prepare students to take up postings to the rural communities’. HF2MD. And: ‘By allowing medical students to visit and possibly spend more time in rural areas during their academy and clinical work helps them to prepare themselves willingly to work in rural areas’. FH4MD.*

##### Introduction of community-based education into the curriculum

In much the same vein as the previous point, participants made a case for introducing community-based education into medical training. This would expose students to the living conditions and lifestyle of the rural communities and help them appreciate the plight of the rural people. As a result, they would be better able to cope with rural life when posted there after graduation.

*‘COBES, which was part of my medical training, exposed me to the rural areas where I understood the plight of the rural folk’. HF10MD. And: ‘UDS-SMHS graduates are doing well in the regions and should be recommended to other universities’. HF6MD.*

##### Regular assessment of the needs of rural doctors

Respondents further suggested that conducting regular research activities which seek the opinion of doctors on what motivates them to serve or leave the rural community, could inform the recruitment of doctors to rural areas and their retention for policymakers’ consideration: *‘By organising regular surveys among doctors in the rural areas to identify their needs and presenting such results to the authorities concerned’. HF1MD.*

#### Collaboration between the key players

The final suggestion put forward by participants was that key players would do well to collaborate on matters of rural practice. For instance, medical schools should work together in partnership with the community in order to render its COBES programme effective. Such collaboration could result in the community offering transportation and supervisory assistance to students. It would also create a favourable learning environment for students, which, in turn, could increase the likelihood of them returning to the rural areas in the future: *‘If the medical schools can collaborate with the rural hospitals in terms of them accepting medical students during their community postings as well as treating them well during their stay in the communities (COBES)’. HF10MD.*

##### Accreditation of district hospitals for training

Participants also suggested that schools, hospitals and national authorities should join forces in order to increase the number of accredited hospitals in the region. By sending specialists to some of the district hospitals, the Ministry of Health could help raise quality standards in these institutions, making them eligible for accreditation. As a result, students would have more opportunities to undertake an accredited specialty training after graduation. A positive experience could make young doctors want to stay and work there after their training has ended, thereby helping address the undersupply of doctors in such areas: *‘We need more district hospitals to be accredited for training to enhance compulsory posting of interns to the district hospitals’. HF1MD.*

## Discussion

A number of factors influence medical doctors’ decision to practise in rural remote communities. The medical doctors who took part in this study had accepted rural postings for the opportunity it afforded them to take an active role in patient care, gain experience and hone their clinical skills, and also to ultimately gain professional independence. Apart from these reasons, they were drawn by a desire to serve the needy in a completely new environment. These results echo and confirm previous international research findings suggesting that acquiring experience, clinical skills and professional independence are common reasons for doctors to choose rural practice [6, 7, 17–19]. Although the doctors in this study had gravitated to remote areas on their own free will, they were challenged, negatively, by the lack of adequate infrastructure and social amenities in the rural areas. This is a common problem in Ghana and middle- and low-income countries in sub-Saharan Africa. Lack of suitable accommodation, inadequate basic tools and equipment were very disheartening and frustrated these doctors in the rural communities. According to participants, part of the solution to these community problems lay in effective collaboration between the community, medical schools and district hospitals. For instance, if the medical schools would clearly communicate to the district hospitals the time at which the students would arrive for their COBES rotation, the hospitals, in collaboration with the community members, could make appropriate accommodation arrangements. Furthermore, the international literature is quite unanimous in suggesting that the absence of better living conditions in rural remote communities, particularly in low- and middle-income countries, adversely affects the retention of health workers, particularly doctors, in remote areas [6–8].

Lack of career progression opportunities and professional isolation were key complaints voiced by the doctors in this study. Despite their willingness to work in rural areas, their enthusiasm had become overshadowed by a belief that entry into postgraduate studies would have been easier had they stayed in the cities. In their experience, the relevant authorities tended to forget about doctors stationed in the rural area when deciding on allocation of positions for postgraduate medical training. Hence, lack of career progression opportunities became a critical disincentive for these doctors to serve in rural areas. Study delays, lack of continuing professional development and a feeling of being ‘abandoned’ by the ministry were sources of concern to the doctors working in the rural areas. These have been long-standing problems in Ghana and successive governments have been struggling to address some of these challenges [14, 20]. To improve the supply of doctors in rural communities, the government should address these challenges or barriers considering their effects on the attraction and subsequent retention of doctors in the rural areas. This calls for a long-term, attractive and enforceable national policy on rural posting with a strong political commitment and leadership and some collaborative efforts between Ministries of Education and Health and the communities.

An additional key finding in this study is the important and paramount role of medical education. Most of the doctors we surveyed were graduates of the UDS-SMHS which includes rural exposure as an integral part of the curriculum through its PBL/COBES component. To increase the attraction and retention of medical doctors in remote practice in sub-Saharan Africa and middle- and low-income countries, medical students should be exposed to the rural communities early in training. Respondents suggested that medical schools should thus incorporate rural outreach/practice in the curricula to make students spend time in the rural communities before they graduate, in order to prepare them for rural practice and increase the likelihood of them accepting rural postings. The respondents envisaged a clear role for medical education in attracting doctors to such areas and retaining them by means of early rural exposure. To accomplish this, medical schools must incorporate appropriate teaching methods in the curricula, such as COBES, rural rotations and outreaches. Similar suggestions on the role of medical education in the attraction of doctors to the rural communities and their retention have been made by prior studies on Community-Based Education and Service (COBES) [21]. Another finding of this study was that providing students with community-based education may increase their likelihood of working in rural areas after graduation. Similar to previous reports more than 60% of the participants of this study were doctors from UDS-SMHS that runs a community-based programme for students. As shown in a study conducted in Kenya, clinical rotations in peripheral, non-tertiary hospitals motivated students to accept postings in peripheral hospitals [19]. Another study conducted in Uganda also reported that the decision to work in remote and underserved communities was mostly influenced by exposure to rural-based training [22]. It has indeed been reported that students were more inclined to select rural practice upon graduation [23] when they had been previously exposed to rural rotations, a study from Australia reports. Furthermore, a South African study reported that curricular interventions appeared to influence the choice of practice location and that, after controlling for other factors such as rural background and targeted selection of rural students, doctors who worked in rural areas were significantly more likely than urban doctors to report that rural exposure had influenced their practice location [24].

UDS-SMHS graduated doctors previously showed their willingness to work in the rural communities [25]. This is a manifestation of students’ intentions, stated during their medical training and the choice of actual practice location after graduation. Strategies to enhance rural practice call for a review of the content and structure of medical curricula and the introduction of innovative pedagogy that incorporates community-based training as part and parcel of the overall education of medical students.

Another prominent finding in this study was the need to improve basic essential equipment, accredit various district health facilities and post specialists to these facilities for internship training and rural rotations. It is therefore very important for the Ghana Ministry of Health (MoH) to provide the necessary human and material resources for the health facilities to improve the delivery of healthcare in the rural communities. Again, the Ministry of Health, should consider creating incentives packages to attract and retain health professionals in the country and deprived communities in particular. This could be another powerful strategy to retain doctors in the rural areas after their internship. Most of the respondents further believed that the government has a duty to develop human resource policies and plans and should have the political will to implement and monitor them. Curriculum designers can learn from the findings of this and similar studies that instituting Community-Based Education and Service as part of health training institutions’ curricula to provide rural exposure serves the purpose of motivating students and thereby creates a favourable attitude towards rural practice and positively influences graduates’ willingness to work in the rural areas.

### Limitations

This study has some limitations. The study was conducted in only one region of Ghana where UDS-SMHS is located and the limited number of responses to the survey, thus making it difficult to generalise the findings. However, the findings are interesting and concur with similar studies conducted in both developed and developing countries especially on the role of medical education in the training of doctors. The location of UDS-SMHS in the region where the research was conducted could also have influenced the large proportion of UDS-SMHS trained doctors who participated in the study. The regions in which health professions institutions are located generally have more of the professionals practising in those regions. We would welcome replications of the present research in other regions of the country. Another limitation of this study is the use of open-ended questions that provided limited space for participants to write their comments. This did not allow participants to fully express their opinions and perspectives. However, the findings provide valuable information that is useful for future research and also adds to our understanding of the factors that motivate doctors to accept rural practice.

Another limitation is that doctors’ work schedules did not allow for interviews to be conducted as they frequently indicated that their workload was high and they could not free time for the provision of verbal data. Although, the authors have the impression saturation was reached, it cannot be excluded that additional themes would have surfaced in a larger sample. This is a limitation of the study.

## Conclusions

Ghanaian medical doctors are willing to work in the rural areas to acquire clinical skills, experience and professional independence. The key role of medical education in the attraction and retention of doctors to the rural communities was clearly acknowledged. Medical schools should incorporate rural outreach/practice, such as COBES, in the curricula to immerse students in rural practice, increasing the likelihood of them accepting rural postings after graduation.

In addition, the Ministries of Education and Health, in collaboration with the communities could improve the inadequacy of medical doctors in the rural communities by developing a national policy on rural posting. This Policy could be directed towards improvement of district health facilities as centres for internship training and rural rotation for medical doctors.

## Additional file

Additional file 1: Questionnaire. (DOCX 18 kb).

## Abbreviations

CHPS: Community-Based Health Planning and Services
COBES: Community-Based Education and Service
KNUST: Kwame Nkrumah University of Science and Technology
MOE: Ministry of Education
MOH: Ministry of Health
PBL: Problem-Based Learning
SMHS: School of Medicine and Health Sciences
SMS: School of Medical Sciences
TTHERC: Tamale Teaching Hospital Ethical Review Committee
UDS: University for Development Studies
UGSMD: University of Ghana School of Medicine and Dentistry
