# Supplementary material for: Working among the rural communities in Ghana - why doctors choose to engage in rural practice
Source: BMC Med Educ. 2018 Jun 8;18:133. doi: 10.1186/s12909-018-1234-y (PMC5994092; doi:10.1186/s12909-018-1234-y)
Supplement: Supplementary file 1 — Questionnaire. (DOCX 18 kb). [file 12909_2018_1234_MOESM1_ESM.docx]

**UNIVERSITY FOR DEVELOPMENT STUDIES**

**SCHOOL OF MEDICINE AND HEALTH SCIENVES, TAMALE**


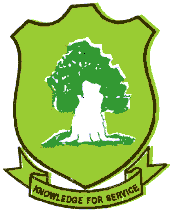


My name is Anthony Amalba of the School of Medicine and Health Sciences of the University for Development Studies, Tamale. I am conducting a research on the Influence of PBL with COBES as an integral part of the undergraduate curriculum on specialty and rural workplace choices. This research is only for academic purposes for the award of PhD. I hereby crave your indulgence to support me in this research by filling the attached questionnaire.

Please, by accepting to complete this questionnaire, it suggests you have consented to participate in the research. Your participation in this study is voluntary and your responses will be accorded the needed confidentiality and anonymity.

Please you are kindly requested to answer all the questions and do so with all sincerity and honesty.

Thank you very much.

Questionnaire

1. Age:----------------------------
2. Sex: 1. Male 2. Female
3. Which Medical school did you graduate from? -------------------------------------------
4. Which year did you graduate?----------------------------------------------------------------
5. How long have you been working in the rural areas? -------------------------------------
6. What motivated you to work in the rural areas? --------------------------------------------------------------------------------------------------------------------------------------------------------------------------------------------------------------------------------------------------------------------------------------------------------------------------------------------------------------------------
7. Has your motivation changed since you started work in the rural areas? 1. Yes 2. No
8. If yes, why-------------------------------------------------------------------------------------------------------------------------------------------------------------------------------------------------------------------------------------------------------------------------------------------------------------------
9. If no, why not? What else is keeping you in the rural area besides reasons given above (Q 6. .c)?------------------------------------------------------------------------------------------------------------------------------------------------------------------------------------------------------------------------------------------------------------------------------------------------------------------------------------------------------------------------------------------------------------------------------------
10. In your opinion why are doctors/health workers in general reluctant to work in rural Ghana (three most important reasons)?--------------------------------------------------------------------------------------------------------------------------------------------------------------------------------------------------------------------------------------------------------------------------------------------------------------------------------------------------------------------------------------------------------------------------------------------------------------------------------------------------------
11. Do you think medical school can prepare medical graduates for rural practice? 1. Yes 2. No
12. If, yes why?------------------------------------------------------------------------------------------------------------------------------------------------------------------------------------------------------------------------------------------------------------------------------------------------------------------
13. If, No why not?--------------------------------------------------------------------------------------------------------------------------------------------------------------------------------------------------------------------------------------------------------------------------------------------------------------
14. In your opinion, did your medical training adequately prepare you for a career in rural practice? 1. Yes 2.No
15. If yes in what ways did it prepare you for a career in rural practice? What were the aspects of the training you valued in this regard?---------------------------------------------------------------------------------------------------------------------------------------------------------------------------------------------------------------------------------------------------------------------------------------------------------------------------------------------------------------------------------
16. If no, why do you think that your medical training did not prepare you for rural practice? What aspects of training would have been contributory from your perspective? -------------------------------------------------------------------------------------------------------------------------------------------------------------------------------------------------------------------------------------------------------------------------------------------------------------------------------------------------------------------------------------------------------------------------------------------------------
17. Are medical students aware of challenges of living and working in rural areas?

1. Yes 2. No

1. If Yes, What are the challenges?

--------------------------------------------------------------------------------------------------------------------------------------------------------------------------------------------------------------------------------------------------------------------------------------------------------------------------------------------------------------------------------------------------------------------------------------------

1. If no, then how, in your opinion, can this awareness be created in the medical curriculum?-----------------------------------------------------------------------------------------------------------------------------------------------------------------------------------------------------------------------------------------------------------------------------------------------------------------------------------------------------------------------------------------------------------------------------
2. What are some of the challenges/barriers one may face when working in rural Ghana as a medical doctor? ----------------------------------------------------------------------------------------------------------------------------------------------------------------------------------------------------------------------------------------------------------------------------------------------------------------------------------------------------------------------------------------------------------------------
3. How can these challenges/barriers regarding rural work place choice of doctors be addressed? ------------------------------------------------------------------------------------------------------------------------------------------------------------------------------------------------------------------------------------------------------------------------------------------------------------------------------------------------------------------------------------------------------------------------------
4. How, in your opinion, can the government encourage doctors to serve in rural Ghana?

--------------------------------------------------------------------------------------------------------------------------------------------------------------------------------------------------------------------------------------------------------------------------------------------------------------------------------------------------------------------------------------------------------------------------------------------

1. Any other comments:------------------------------------------------------------------------------------------------------------------------------------------------------------------------------------------------------------------------------------------------------------------------------------------------------------------------------------------------------------------------------------------------------------------
